# Tribenzotriquinacenes bearing three peripheral or bridgehead urea groups stretched into the 3-D space

**DOI:** 10.3762/bjoc.7.43

**Published:** 2011-03-18

**Authors:** Jörg Tellenbröker, Dietmar Kuck

**Affiliations:** 1 Department of Chemistry, Bielefeld University, Universitätsstraße 25, D-33615 Bielefeld, Germany

**Keywords:** convex–concave structures, polycyclic compounds, supramolecular chemistry, tribenzotriquinacenes, urea derivatives

## Abstract

The syntheses of tribenzotriquinacenes (TBTQ) bearing three phenylurea groupings at either the arene periphery or at the benzhydrylic bridgeheads of the rigid, convex–concave, *C*_3_*_v_*-symmetrical molecular framework are reported. ^1^H NMR data point to supramolecular aggregation of these TBTQ derivatives in low-polarity solvents.

## Introduction

**Figure 1:**
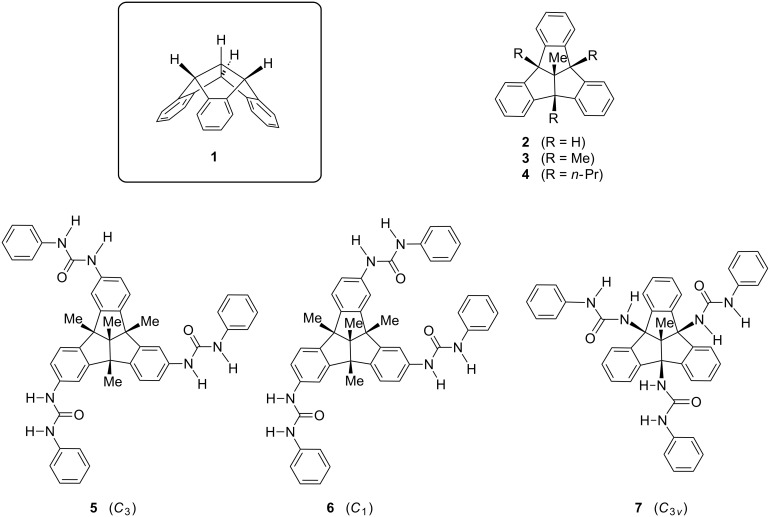
Parent TBTQ **1**, three bridgehead-alkylated congeners **2**–**4**, and three TBTQ tris-urea derivatives **5**–**7** bearing the functional groups in three different spatial orientations.

In the course of our extended research on polyfunctionalized derivatives of tribenzotriquinacene (TBTQ, **1**) and some hydrocarbon congeners **2**–**4**, which provide a great variety of convex–concave molecular building blocks [1–7], we focused our attention on the synthesis of TBTQ derivatives **5**–**7** with three urea units located at the molecular periphery (Figure 1). Owing to the rigidity of their molecular framework, these compounds were considered of potential interest for the formation of supramolecular aggregates consisting of two or more multiply hydrogen-bonded TBTQ units. Research on the formation and chemical properties of host–guest compounds bearing urea units as linker groups has become an exciting field of scientific interest over the past few decades [8–15]. For example, cone-like building blocks such as those based on calix[4]arenes and calix[6]arenes bearing urea groups at their lower rims have been used for the generation of multi-hydrogen bonded dimers [8–9]. Therefore, the TBTQ framework, with its close similarity to that of the cyclotribenzylenes and the cyclotriveratrylenes [16–19], should constitute a promising substrate for such studies. In the present paper, we wish to report the facile access to TBTQs that bear three phenylurea groups directed into the 3-D space in well controlled orientations. Some observations that point to non-covalent aggregation of these novel molecules are also reported.

## Results and Discussion

**Scheme 1:**
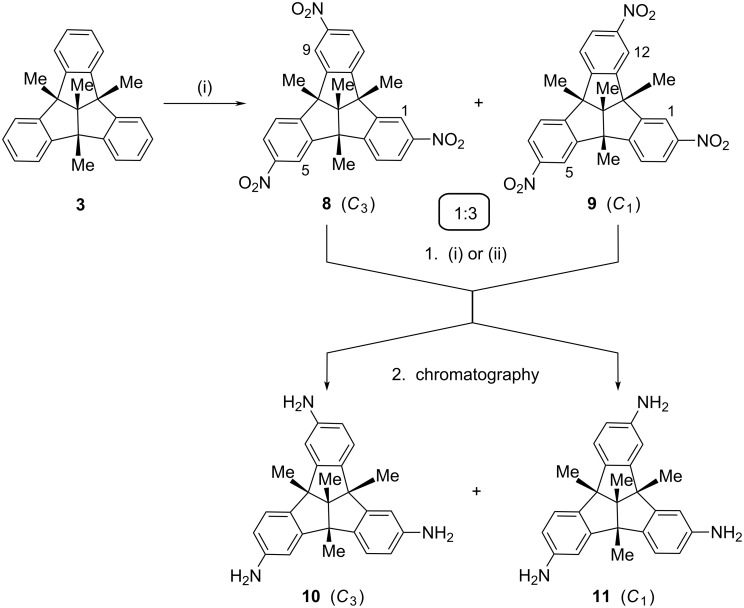
Nitration of tetramethyl-TBTQ **3** to give a 1:3 mixture of the trinitro derivatives **8** and **9**, and subsequent reduction of the mixture, yielding the TBTQ triamines **10** and **11**. Reagents, conditions and yields: (i) NaNO_3_, TFA, 25 °C, 48 h, quantitative. (ii) N_2_H_4_ · H_2_O (96%), FeCl_3_ · 6 H_2_O, charcoal, 80 °C, 24 h, 81%. Alternatively: (ii) H_2_, Pd/C, EtOH, 5 bar, 20 °C, 24 h, 91%; yields after chromatography 20% (**10**) and 70% (**11**).

As shown earlier, introduction of six amino groups at the periphery of the TBTQ skeleton is achievable by nitration with nitric acid (100%) and sulfuric acid (98%) followed by reduction [20]. However, this holds true only for TBTQ derivatives with alkyl groups at the benzhydrylic bridgehead positions, such as the tetramethyl derivative **3** [20] and the related tripropyl analog **4** [5,7]. The monomethyl derivative **2** decomposes under these conditions. By contrast, threefold nitration of the arene periphery, leading to single functionalization at the outer positions of each of the three benzene rings, was achieved by use of sodium nitrate in trifluoroacetic acid [21]. As shown in Scheme 1, this method allowed us to convert the tetramethyl derivate **3** into a mixture of the *C*_3_-symmetrical compound **8** and the *C*_1_-symmetrical isomer **9** in apparently quantitative yield. More recently, this method was also successfully applied to the analogous nitration of compound **2** [22].

Our attempts to separate the two constitutional isomers **8** and **9** by gravity column chromatography failed due to their almost identical elution behaviour. However, ^1^H NMR spectroscopy of the mixture allowed us to determine the ratio of the isomers in the crude reaction mixture. As expected, the symmetrical compound **8** was the minor component and the ratio observed, **8**:**9** ≈ 1:3, suggested a random attack of the electrophile at each of the benzene rings even in the second and third step of the threefold nitration. This corresponds to comparable findings with multiple electrophilic substitutions of the centropolyindanes and reflects the lack of electronic interaction between the aromatic units [22].

**Figure 2:**
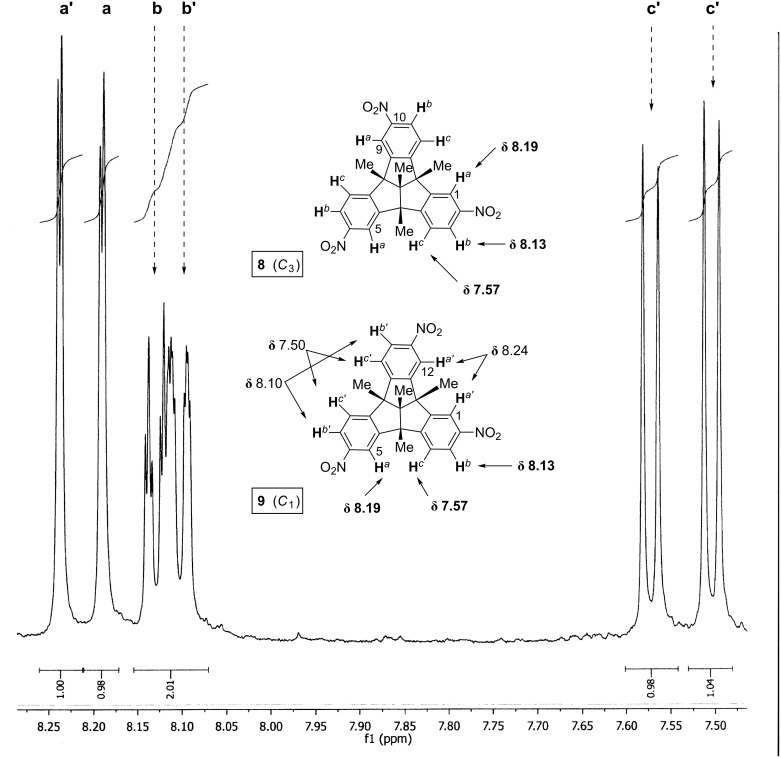
Partial ^1^H NMR spectrum (500 MHz, CDCl_3_) of the mixture of the trinitro-TBTQ isomers **8** and **9**.

Because of the prototypical character of three-fold nitration of a TBTQ derivative, the ability to distinguish between the *C*_3_- and the *C*_1_-symmetrical isomers **8** and **9** and to assess the relative amounts is addressed here in some detail (Figure 2). Notwithstanding the fact that the ^1^H NMR spectra of compounds **8** and **9** are very similar, their isomer ratio can be determined from the mixture by assuming incremental deshielding effects caused by the nitro group placed at the benzene ring which is adjacent to that bearing the proton under observation. Thus, besides the strong deshielding effect due to the *ortho-*NO_2_ groups of the very same benzene ring, the “distal” or “proximal” nitro group at the closest neighbouring ring affects the chemical shift. For example, the resonance of the (“isolated”) *ortho*-proton 1-H of isomer **8** (H*^a^*, δ 8.19) is also affected to a minor extent by the distal 10-NO_2_ group. The equivalent protons 5-H*^a^* and 9-H*^a^* of **8** suffer the same extra chemical shift but, interestingly, the isolated proton 5-H*^a^* of isomer **9** is also isochronous and contributes to the doublet (^4^*J* = 2.0 Hz) at δ 8.19. By contrast, the remaining isolated *ortho*-protons, 1-H and 12-H, of isomer **9** resonate at slightly lower field (H*^a’^*, δ 8.24) due to the stronger deshielding effect of the proximal 11-NO_2_ and 2-NO_2_ groups, respectively. Correspondingly, this significant difference in the chemical shifts (Δδ = 0.05 ppm) of protons H*^a^* and H*^a’^* of isomers **8** and **9** is found to occur inversely with the resonances of the non-isolated *ortho*-protons H*^c^* and H*^c’^*, and appear as doublets (^3^*J* = 8.5 Hz) at δ 7.57 and δ 7.50, respectively. Thus, the former resonance is assigned to the equivalent protons 4-H*^c^*, 9-H*^c^* and 12-H*^c^* of **8** and the isochronous proton 4-H*^c^* of **9**, all having one proximal nitro group at the adjacent benzene ring. The latter resonance assigned to the isochronous protons 8-H*^c’^* and 9-H*^c’^* is slightly upfield-shifted (Δδ = 0.07 ppm) due to the weaker deshielding effect of the respective distal 11-NO_2_ and 6-NO_2_ groups. Similar arguments hold true for the *meta*-protons H*^b^* and H*^b’^* of the TBTQ skeleton, with a noticeably slight perturbation in the case of H*^b^* due to the asymmetry of **9**. In fact, the unity integral ratios observed for the six different resonances *a* and *a’*, *b* and *b’*, and *c* and *c’* are in excellent agreement with the assumed isomer ratio **8**:**9** = 25:75: With this ratio, the integrals of the groups of isochronous protons, e.g., H*^a^* in **8** and **9** and H*^a’^* in **9**, become equal: [H*^a^*] = 0.25 × 3 + 0.75 × 1 = [H*^a’^*] = 0.75 × 2 = 1.50. Finally, it may be noted that, accordingly, the 1:3 mixture of the trinitro compounds **8** and **9** is also reflected by three ^13^C NMR lines at δ 25.54, 25.46 and 25.40 in the intensity ratio of ca. 3:6:3.

Reduction of the mixture of the trinitro compounds **8** and **9** was in the first instance achieved by use of hydrazine with iron(III) chloride and charcoal in refluxing methanol (Scheme 1) [23], and afforded a mixture of the triamino-TBTQs **10** and **11** in good yield (81%). However, catalytic hydrogenolysis of compounds **8** and **9** at ambient temperature under medium pressure [20,24–25] proved to be even more efficient and gave the TBTQ-trianilines **10** and **11** in an even higher yield (91%). At variance from the trinitrotribenzotriquinacenes, these derivatives were found to display quite different elution behaviour on silica gel. By use of ethyl acetate as an eluent, the retention factors of **10** and **11** were found to be 0.44 and 0.27. After separation by column chromatography, the first-eluting, *C*_3_-symmetrical isomer **10** was obtained in 20% and the second-eluting, *C*_1_-symmetrical isomer **11** in 70% yields, in line with the isomer ratio deduced for the trinitro precursors.

Similar to the trinitro compounds **8** and **9**, the triamines **10** and **11** were found to give apparently identical EI mass spectra. In all of these cases, loss of a methyl radical is, by far, the dominating primary fragmentation process. However, ^1^H and ^13^C NMR spectroscopy allowed us to unequivocally assign the isomers. The *C*_3_-symmetry of compound **10** is reflected by the relatively well resolved doublets at δ 6.47 and 7.06 and the relatively sharp singlet at δ 6.59, all of which having unity integral ratios. As expected, the chemical shifts in the spectrum of the *C*_1_-symmetrical isomer **11** are very similar to those of **10** but very slight differences of the chemical shifts are evident from the significantly broadened and more complex signals. Notably, these differences are much less pronounced as was the case with the trinitro precursors; thus, the splitting of the resonances of the isolated protons 1-H, 5-H and 12-H of isomer **11** into two singlets at δ 6.59 (1H) and δ 6.61 (2H) is minute. Moreover, and quite specifically, the proton-decoupled ^13^C NMR spectrum of the *C*_3_-symmetrical isomer **10** exhibits a single line at δ 61.7 due to the three benzhydrylic bridgeheads and another line at δ 25.9 due to the three methyl groups attached to these positions, whereas the spectrum of **11** shows two sets of three lines at δ 61.1, 61.7 and 62.2 and at δ 25.7, 25.9 and 26.1, respectively.

**Scheme 2:**
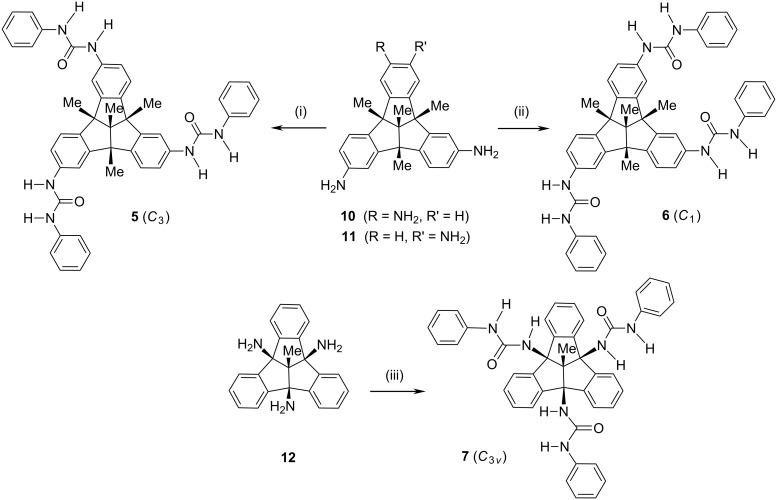
Syntheses of tris-phenylurea-substituted tribenzotriquinacenes **5**–**7**. Reagents and conditions: (i) PhNCO, CHCl_3_, 50 °C, 6 h, 84%. (ii) ditto, 86%. (iii) ditto, 96%.

The triamino-substituted TBTQs **10** and **11** readily added three equivalents of phenylisocyanate to give the corresponding *C*_3_- and *C*_1_-symmetrical TBTQ-based tris-ureas **5** and **6** in very good yields (Scheme 2). These conversions were as efficient as the corresponding reactions of the tetraaminocalix[4]arenes [10]. As expected, the colorless amorphous solids had good solubility in polar solvents but not in non-polar ones. Whereas EI and DEI mass spectrometry failed, ionization by FAB(+) furnished almost identical mass spectra that clearly showed the expected [M + H]^+^ peaks at *m*/*z* 739 along with very minor signals for the twofold condensation product. However, the ^1^H NMR spectra of compounds **5** and **6** gave no hint to such impurities.

In analogy to the triamino precursors **10** and **11**, the TBTQ-tris-ureas **5** and **6** gave very similar ^1^H NMR spectra even at 600 MHz but, again, the spectrum of the non-symmetrical **6** isomer revealed significant splitting of the peaks. The spectrum of the *C*_3_-symmetrical **5** isomer exhibited two characteristic singlets at δ 8.54 and δ 8.60 for the two sets of amido protons. ^1^H,^1^H-COSY measurements allowed us to assign these resonances to the “outer” (Ph–N*H*CO) and the “inner” (TBTQ–N*H*CO) amido protons, respectively, of the three equivalent urea groups. In fact, the singlet for the adjacent protons, 1-H, 5-H and 9-H (δ 7.55), at the TBTQ nucleus, as well as the doublet for the six *ortho*-protons of the phenyl groups (δ 7.43), were found to be useful for this assignment. The three equivalent bridgehead methyl groups of **5** resonate at δ 1.57 and those of the central methyl group appear at δ 1.30.

Although the bridgehead chemistry of the tribenzotriquinacenes has been extensively investigated [1–4,7], the attachment of multifunctional groupings at the convex surface of the TBTQ skeleton has been only scarcely examined [4]. Therefore, we subjected the previously described bridgehead triaminotribenzotriquinacene **12** [4] to the reaction with phenylisocyanate in analogy to the conversion of the peripheral triamines **10** and **11**, and isolated the corresponding *C*_3_*_v_*-symmetrical TBTQ tris-urea **7** in very good yield (Scheme 2). Again, characterization by FAB(+) and also by ESI(+) mass spectrometry was straightforward; intense [M + H]^+^ (*m*/*z* 697) and, respectively, [M + Na]^+^ (*m*/*z* 719) peaks were in accord with the product of threefold addition. Interestingly, the ^1^H NMR spectra of compound **7** showed pronounced solvent effects on the NH proton resonances. The outer protons were found to resonate at δ 8.86 in DMSO-*d*_6_ but at δ 6.80 in C_2_D_2_Cl_4_, and the inner protons to resonate at δ 6.57 in DMSO-*d*_6_ and at δ 5.37 in C_2_D_2_Cl_4_. Thus, the change from the polar to the low-polarity solvent gives rise to a high-field shifts of Δδ = 2.3 and 1.4 ppm, respectively. This effect was attributed to the preferred formation of hydrogen-bound dimers of the tris-urea **7** in tetrachloroethane solution.

As outlined above, the potential dimerization of the threefold urea-functionalized TBTQ derivatives **5**, **6** and **7** was one motif for this work. In fact, some evidence for the formation of neutral dimers in low-polarity solvents was found. As mentioned above, the strong high-field shift observed in dichloroethane solution for the NH protons of the bridgehead TBTQ tris-urea **7** suggests the formation of a dimeric aggregate. Specifically, the particularly strong shielding of the external NH protons (Δδ = −2.3) may point to the formation of face-to-face dimers in which the arene units of the opposite TBTQ core exert pronounced magnetic anisotropy effects.

In the case of the peripheral TBTQ tris-ureas **5** and **6**, ^1^H NMR measurements in DMSO and tetrachloroethane revealed a surprising effect which may also be attributed to aggregation of these molecules in low-polarity solvents. Both of these compounds were found to be only poorly soluble in C_2_D_2_Cl_4_ and their ^1^H NMR spectra in this solvent showed very broad signals for the NH protons. Therefore, assignment of NH and arene proton resonances was impossible even at elevated temperatures (≤ 120 °C). Nevertheless, the ^1^H resonances of the three benzhydrylic and the single central methyl groups were clearly detectable in both DMSO-*d*_6_ and C_2_D_2_Cl_4_. However, in the latter solvent, the protons of the benzhydrylic methyl groups of **5** and **6** were found to resonate at significantly higher fields than those of the central methyl group (Table 1). This is in stark contrast to the relative chemical shifts found with DMSO-*d*_6_ as a solvent and with all TBTQ derivatives studied so far.

**Table 1:** Solvent effect on the chemical shifts (δ) observed in the ^1^H NMR spectra (500 MHz) of the central (12d-) and the outer bridgehead (4b-, 8b-, 12b-) methyl groups of compounds **5** and **6**.

Compound	Bridgehead methyl groups	Solvent	
		DMSO-*d*_6_	C_2_D_2_Cl_4_

**5**	12d-CH_3_	1.30	1.34
	4b-, 8b-, 12b-CH_3_	1.57	1.17
**6**	12d-CH_3_	1.30	1.26
	4b-, 8b-, 12b-CH_3_	1.56	1.07

It is noteworthy that the ^1^H resonance of the central methyl group is hardly affected by the solvent, whereas the resonance of the outer bridgehead methyl groups is shifted to a higher field by Δδ ≈ 0.4 ppm for the *C*_3_-symmetrical compound **5** and by even Δδ ≈ 0.5 ppm for the *C*_1_-symmetrical compound **5**. Similar to the argument mentioned above for compound **7**, this effect could be attributed to the shielding effect exerted by the arene units of the TBTQ core belonging to the opposite molecule associated in face-to-face dimers of either **5** or **6**. Notably, however, and in contrast to the case of compound **7**, the TBTQ molecules in such dimers would be aggregated with their concave sides toward each other.

Finally, it should be noted that all of the FAB(+) and ESI(+) mass spectra recorded with compounds **5**, **6** and **7** exhibited strong signals for dimeric quasi-molecular ions. For example, the ESI(+) mass spectrum of the *C*_1_-symmetrical isomer **6** obtained from pure methanol solution exhibited the ions [M + Na]^+^ (*m*/*z* 761) and [2 M + Na]^+^ (*m*/*z* 1499) in the abundance ratio of ca. 100:20. About the same ratio was found when a chloroform/methanol mixture (99:1) was used. By contrast, the *C*_3_-symmetrical isomer **5** sprayed from the latter solvent gave ESI(+) mass spectra that reproducibly show abundant peaks for (singly charged) [M + 2 Na]^+^ (*m*/*z* 784) and [2 M + 2 Na]^+^ ions. This difference may be due to the different molecular symmetry. The FAB(+) mass spectrum of the bridgehead tris-urea **7** (obtained with NBA as a matrix) and the corresponding ESI(+) mass spectrum obtained from pure methanol exhibited the [M + H]^+^ (*m*/*z* 697) and the [M + Na]^+^ (*m*/*z* 719) peaks, respectively, but only very minor dimeric quasi-molecular ion peaks.

No more detailed analysis of the dimerization of the new TBTQ-based tris-urea **5**, **6**, and **7** has been carried out so far. Also, it remains open whether homo- or heterochiral association is preferred with the peripheral tris-urea **5** and **6** and whether the *C*_3_-symmetrical isomer **5** behaves differently from the *C*_1_-symmetrical isomer **6**. Nevertheless, the observations reported in the present work point to the existence of TBTQ tris-urea dimers in non-polar solvents. Congeners bearing three longer-chain aliphatic groups in place of the phenylcarbamoyl residues appear to be good candidates for further studies in this field.

## Experimental

**2,6,10-Trinitro-4b,8b,12b,12d-tetramethyl-4b,8b,12b,12d-tetrahydrodibenzo-[2,3:4,5]pentaleno[1,6-*ab*]indene (8) and 2,6,11-trinitro-4b,8b,12b,12d-tetramethyl-4b,8b,12b,12d-tetrahydrodibenzo[2,3:4,5]-pentaleno-[1,6-*ab*]indene (9) (mixture of isomers).** A suspension of tetramethyltribenzotriquinacene **3** (250 mg, 743 mmol) in trifluoroacetic acid (40 mL) was stirred while sodium nitrate (756 mg, 8.92 mmol) was added in small portions. The reaction mixture became a yellow solution after several hours and stirring was continued for a total of 48 h at ambient temperature. After the addition of water (25 mL), the mixture was cooled with ice and the pH adjusted to ≈10 by the careful addition of sodium hydroxide (solid or 6 M aqueous solution). Repeated extraction with chloroform, drying of the combined extracts with sodium sulfate and removal of the solvent under reduced pressure followed by column chromatography (silica gel/CHCl_3_) gave a mixture of the trinitro compounds **8** and **9** (350 mg, 100%) in a ratio of ca. 1:3 as a yellow, amorphous solid; *R*_f_ (CHCl_3_) 0.74; mp >360 °C; IR (KBr): ν = 3073, 2974, 2934, 1594, 1530, 1475, 1453, 1396, 1345, 1207, 1153, 1112, 1097, 1067, 1032, 907, 887, 825, 742, 674 cm^−1^; ^1^H NMR (500 MHz, CDCl_3_), integral data given as parts of a total of 9 H*^ar^* and 12 H*^me^*: δ = 8.240 (d, *J* = 2.1 Hz, 1.5 H*^ar^*), 8.193 (d, *J* = 2.1 Hz, 1.5 H*^ar^*), 8.125 (d, *J* ≈ 8.5 Hz, with fine splitting of *J* ≈ 1.9 Hz, 1.5 H*^ar^*), 8.105 (br d, *J* ≈ 8.5 Hz, 1.5 H*^ar^*), 7.575 (d, *J* = 8.5 Hz, 1.5 H*^ar^*), 7.506 (d, *J* = 8.5 Hz, 1.5 H*^ar^*), 1.77 (s, 2.25 H*^me^*), 1.75 (s, 4.5 H*^me^*), 1.73 (s, 2.25 H*^me^*), 1.43 (s, 3H*^me^*); ^13^C NMR (75 MHz, acetone-*d*_6_): δ = 155.9, 155.6, 150.8, 150.7, 149.9 (all C), 125.6, 124.8, 124.7, 119.7 (all CH), 72.3, 64.1, 63.94, 63.86 (all C), 25.54, 25.46, 25.4, 16.1 (all CH_3_); MS (EI, 70 eV) *m*/*z* (%): 471 (9) [M^+•^], 456 (100), 410 (9), 395 (7); accurate mass (EI, 70 eV): *m*/*z* 456 ([M – CH_3_]^+^), C_25_H_18_N_3_O_6_, calcd 456.1196; found 456.1196.

**2,6,10-Triamino-4b,8b,12b,12d-tetramethyl-4b,8b,12b,12d-tetrahydrodibenzo-[2,3:4,5]pentaleno[1,6-*ab*]indene (10) and 2,6,11-triamino-4b,8b,12b,12d-tetramethyl-4b,8b,12b,12d-tetrahydrodibenzo[2,3:4,5]pentaleno[1,6-*ab*]indene (11). *Procedure A.*** A suspension containing the mixture of isomeric trinitrotribenzotriquinacenes **8** and **9** described above (230 mg, 488 mmol), charcoal (32 mg) and iron(III) chloride hexahydrate (10 mg) in methanol (10 mL) was heated to reflux for 15 min and then hydrazine hydrate (97%, 120 mg) added. After heating for 4 h further FeCl_3_•6H_2_O (10 mg) and 120 mg N_2_H_4_•H_2_O (120 mg) were added. The mixture was heated under reflux for a total of 24 h, allowed to cool and filtered through silica with ethyl acetate. Removal of the solvent gave a mixture of the triamines **10** and **11** (151 mg, 81%) as a yellow solid, the components of which can be separated by chromatography. ***Procedure B.*** The mixture of isomers **8** and **9** described above (500 mg, 1.06 mmol) and Pd/C (10%, 100 mg) were suspended in ethanol (100 mL) and shaken under a hydrogen atmosphere at 5 bar at ambient temperature for 24 h. The catalyst was removed by filtration through silica gel and the solvent removed under reduced pressure. The triamines **10** and **11** were separated by column chromatography (silica gel, EtOAc) to yield the *C*_3_-symmetrical isomer **10** (115 mg, 20%) and the *C*_1_-symmetrical isomer **11** (399 mg, 70%). ***Isomer 10*:*****R*_f_ (EtOAc) 0.44; mp 344 °C; IR (KBr): ν = 3445, 3372, 3208, 3013, 2960, 2927, 2866, 1619, 1494, 1449, 1434, 1389, 1370, 1313, 1276, 1258, 1238, 1188, 1157, 1137, 1078, 1028, 926, 857, 815, 756, 708, 613 cm^−1^; ^1^H NMR (500 MHz, CDCl_3_): δ = 7.06 (d, *J* = 8.0 Hz, 3H), 6.59 (s, 3H), 6.47 (d, *J* = 7.4 Hz, 3H), 3.45 (br s, 6H), 1.53 (s, 9H), 1.27 (s, 3H); ^13^C NMR (125 MHz, CDCl_3_): δ = 150.7, 145.9, 139.2 (all C), 123.3, 115.1, 109.1 (all CH), 70.6 (C), 61.7 (C), 25.9 (CH_3_), 16.1 (CH_3_); MS (EI, 70 eV) *m*/*z* (%): 381 (26) [M^+•^], 366 (100), 351 (5), 336 (9), 319 (5); accurate mass (EI, 70 eV): C_26_H_27_N_3_ (M^+•^), calcd 381.2205, found 381.2206. ***Isomer 11*:** *R*_f_ (EtOAc) 0.27; mp 339 °C; IR (KBr): ν = 3454, 3374, 3216, 3014, 2961, 2929, 2864, 1618, 1490, 1453, 1434, 1392, 1371, 1311, 1266, 1245, 1183, 1156, 1133, 1077, 1027, 931, 860, 814, 753 cm^−1^; ^1^H NMR (500 MHz, CDCl_3_): δ = 7.09–7.03 (m, 3H), 6.61 (s, 2H), 6.59 (s, 1H), 6.49–6.42 (m, 3H), 3.41 (br s, 6H), 1.53 (s, 9H), 1.27 (s, 3H); ^13^C NMR (125 MHz, CDCl_3_): δ = 150.3, 150.0, 149.6, 145.8, 145.7, 145.6, 140.3, 139.9, 139.6 (all C), 123.4, 123.22, 123.19, 115.4, 115.3, 115.2, 109.3, 109.1 (all CH), 70.6, 62.2, 61.7, 61.1 (all C), 26.1, 25.9, 25.7, 16.1 (all CH_3_); MS (EI, 70 eV) *m*/*z* (%): 381 (20) [M^+•^], 366 (100), 351 (4), 336 (8), 319 (4); accurate mass (EI, 70 eV): C_26_H_27_N_3_ (M^+•^), calcd 381.2205, found 381.2204.

**2,6,10-Tris[(*N*-phenylcarbamoyl)amino]-4b,8b,12b,12d-tetramethyl-4b,8b,12b,12d-tetrahydrodibenzo[2,3:4,5]pentaleno[1,6-*ab*]indene (5).** A solution of the triamino-TBTQ **10** (115 mg, 302 μmol) in anhydrous chloroform (70 mL) was stirred under an argon atmosphere while phenylisocyanate (108 μL, 995 μmol) was added. The mixture was heated at 50 °C for 6 h and the solvent was removed under reduced pressure. The colorless solid residue was suspended in *n*-hexane, filtered by suction and dried in vacuo to give the tris-urea **5** (187 mg, 84%) as a colorless amorphous solid, mp >360 °C; IR (KBr): ν = 3372, 2968, 2928, 1663, 1598, 1546, 1491, 1443, 1416, 1372, 1311, 1227, 1080, 752, 692 cm^−1^; ^1^H NMR (500 MHz, DMSO-*d*_6_), see Supporting Information File 1 for 600 MHz spectrum: δ = 8.60 (s, 3H), 8.54 (s, 3H), 7.55 (d, *J* = 1.4 Hz, 3H), 7.43 (d, *J* = 8.0 Hz, 6H), 7.26 (t, *J* = 7.9 Hz, 9H), 7.18 (dd, ^3^*J* = 8.4 Hz, ^4^*J* = 1.4 Hz, 3H), 6.94 (t, *J* = 7.4 Hz, 3H), 1.57 (s, 9H), 1.30 (s, 3H); ^13^C NMR (125 MHz, DMSO-d_6_): δ = 152.6, 149.5, 142.1, 139.8, 139.1 (all C), 128.8, 122.8, 121.8, 118.2, 112.6 (all CH), 69.9 (C), 61.7 (C), 25.5 (CH_3_). The 12d-CH_3_ group does not show a separate resonance. ^1^H NMR (500 MHz, C_2_D_2_Cl_4_): δ = 7.30–6.90 (m, 30H), 1.34 (s, 3H), 1.17 (s, 9H); MS (FAB(+), NBA) *m*/*z* (%): 1479 (1) [2 M + H]^+^, 739 (97) [M + H]^+^, 723 (19); MS [ESI(+), MeOH] *m*/*z* (%): 1499.8 (8) [2 M + Na]^+^, 1358.7 (6), 761.3 (100) [M + Na]^+^, 756.4 (19), 739.3 (12), 642.3 (25), 620.3 (28); MS [ESI(+), CHCl_3_ and ~1% MeOH] *m*/*z* (%): 1565.8 (1), 1523.8 (7) [2 M + 2 Na]^+^, 1500.8 (1), 1403.8 (1), 1358.7 (1), 826.4 (25), 784.4 (100) [M + 2 Na]^+^, 757.4 (19), 665.4 (68); MS [ESI(+), MeOH, (CH_3_)_4_NPF_6_] *m*/*z* (%): 1551.9 (2) [2 M + (CH_3_)_4_N]^+^, 812.5 (100) [M + (CH_3_)_4_N]^+^, 761.3 (46), 680.1 (52), 620.3 (12); combustional analysis: C_47_H_42_N_6_O_3_•H_2_O calcd C 74.58, H 5.86, N 11.10, found C 74.57, H 6.09, N 10.98.

**2,6,11-Tris[(*N*-phenylcarbamoyl)amino]-4b,8b,12b,12d-tetramethyl-4b,8b,12b,12d-tetrahydrodibenzo[2,3:4,5]pentaleno[1,6-*ab*]indene (6).** In analogy to the procedure given above, triaminotribenzotriquinacene **11** (104 μg, 273 μmol) was dissolved in anhydrous chloroform (70 mL) and reacted with phenylisocyanate (98 μL, 901 μmol) under argon. The mixture was heated at 50 °C for 6 h and then treated as above to yield the tris-urea **6** (173 mg, 86%) as a colorless, amorphous solid, mp 257 °C (decomp.); IR (KBr): ν = 3381, 2968, 2928, 1664, 1598, 1549, 1491, 1443, 1415, 1372, 1311, 1227, 1079, 752, 692 cm^−1^; ^1^H NMR (500 MHz, DMSO-*d*_6_), see Supporting Information File 1 for 600 MHz spectrum: δ = 8.62 (s, 1H), 8.60 (s, 2H), 8.57 (s, 1H), 8.56 (s, 1H), 8.54 (s, 1H), 7.53 (d, *J* = 1.4 Hz, 1H), 7.49 (d, *J* = 1.3 Hz, 1H), 7.46 (d, *J* = 1.4 Hz, 1H), 7.42 (d, *J* = 7.9 Hz, 6H), 7.38 (d, *J* = 8.4 Hz, 1H), 7.37 (d, *J* = 8.3 Hz, 1H), 7.25 (m, 9H), 7.16 (dd, *J* = 8.3 Hz, *J* = 1.5 Hz, 1H), 6.94 (t, *J* = 7.3 Hz, 3H), 1.56 (s, 9H), 1.30 (s, 3H). ^13^C NMR (125 MHz, DMSO-*d*_6_): δ = 152.6, 149.2, 149.0, 148.7, 142.9, 142.6, 142.4, 139.7, 139.1, 139.0 (all C), 128.7, 123.1, 123.0, 122.9, 121.7, 118.4, 118.2, 112.5, 112.4, 112.3 (all CH), 69.9, 62.2, 61.7, 61.3 (all C), 25.6 (CH_3_), 15.8 (CH_3_); ^1^H NMR (500 MHz, C_2_D_2_Cl_4_): δ = 7.32 (br s), 7.22 (br s), 7.20 (br s), 7.19 (br s), 7.03 (br s), 7.00 (br s), 6.91 (br s), 1.26 (br s, 3H), 1.07 (br s, 9H); MS [FAB(+), NBA] *m*/*z* (%): 1478 (2) [2 M + H]^+^, 739 (73) [M + H]^+^, 723 (7); MS [ESI(+), MeOH] *m*/*z* (%): 1500.6 (19) [2 M + Na]^+^, 1136.4 (4), 767.3 (9), 761.3 (100) [M + Na]^+^, 756.3 (28), 739.3 (12), 670.3 (6), 620.3 (7); MS [ESI(+), CHCl_3_ and ~1% MeOH] *m*/*z* (%): 1500.7 (9) [2 M + Na]^+^, 761.4 (100) [M + Na]^+^; MS [ESI(+), MeOH, (CH_3_)_4_NPF_6_] *m*/*z* (%): 1551.8 (5) [2 M + (CH_3_)_4_N]^+^, 1499.6 (5), 848.6 (17), 812.4 (70) [M + (CH_3_)_4_N]^+^, 761.3 (76), 756.3 (100), 739.3 (54), 663.4 (20), 620.3 (27), 529.4 (30); combustional analysis: C_47_H_42_N_6_O_3_•H_2_O calcd C 74.58, H 5.86, N 11.10, found C 74.51, H 5.92, N 10.92.

**12d-Methyl-4b,8b,12b-tris[(*N*-phenylcarbamoyl)amino]-4b,8b,12b,12d-tetrahydrodibenzo[2,3:4,5]pentaleno[1,6-*ab*]indene (7).** Similar to the procedures given above, triaminotribenzotriquinacene **12** (125 μg, 371 μmol) and phenylisocyanate (133 μL, 1.22 μmol) were reacted in anhydrous chloroform (70 mL) at 50 °C for 6 h and then treated as above to give the tris-urea **7** (246 mg, 96%) as a colorless, amorphous solid, mp 321 °C; IR (KBr): ν = 3397, 3064, 1682, 1631, 1598, 1542, 1498, 1441, 1351, 1311, 1237, 751, 692, 663 cm^−1^; ^1^H NMR (500 MHz, DMSO-*d*_6_), see Supporting Information File 1 for 600 MHz spectrum: δ = 8.86 (s, 3H), 7.56 and 7.24 (*AA’BB’*, 12H), 7.28 (d, *J* = 7.9 Hz, 6H), 7.15 (t, *J* = 7.7 Hz, 6H), 6.84 (t, *J* = 7.4 Hz, 3H), 6.80 (s, 3H), 1.62 (s, 3H); ^13^C NMR (125 MHz, DMSO-*d*_6_): δ = 154.2, 144.6, 140.4 (all C), 128.6, 128.3, 123.5, 120.9, 117.3 (all CH), 77.1 (C), 76.9 (C), 12.9 (CH_3_); ^1^H NMR (500 MHz, C_2_D_2_Cl_4_, 120 °C): δ = 7.49 and 7.26 (*AA’BB’*, 12H), 7.17 (br s, 12H), 7.00 (m, 3H), 6.57 (s, 3H), 5.37 (s, 3H), 1.71 (s, 3H); MS [FAB(+), NBA] *m*/*z* (%): 1394 (2) [2 M + H]^+^, 697 (100) [M + H]^+^, 561 (78), 426 (14); MS [ESI(+), MeOH] *m*/*z* (%): 1415.7 (2) [2 M + Na]^+^, 1394.6 (1), 1274.6 (1), 1072.9 (1), 719.2 (100) [M + Na]^+^, 697.3 (7), 561.2 (15); MS [ESI(+), MeOH, (CH_3_)_4_NPF_6_)] *m*/*z* (%): 1467.7 (1) [2 M + (CH_3_)_4_N]^+^, 1416.7 (1), 1393.7 (1), 770.3 (20) [M + (CH_3_)_4_N]^+^, 719.2 (100), 697.3 (52), 561.2 (90); combustional analysis: C_44_H_36_N_6_O_3_ calcd C 75.84, H 5.21, N 12.06, found C 75.72, H 5.44, N 12.09; accurate mass [ESI(+)]: [C_44_H_36_N_6_O_3_Na]^+^ calcd 719.2747, found 719.2728.

## Supporting Information

File 1 ^1^H NMR spectra including magnifications of compounds **5**, **6** and **7** (600 MHz) and of compounds **8**, **9**, **10** and **11** (500 MHz).
