# Supplementary material for: Tribenzotriquinacenes bearing three peripheral or bridgehead urea groups stretched into the 3-D space
Source: Beilstein J Org Chem. 2011 Mar 18;7:329–37. doi: 10.3762/bjoc.7.43 (PMC3079111; doi:10.3762/bjoc.7.43)

Supporting Information

for

Tribenzotriquinacenes bearing three peripheral or bridgehead urea groups stretched into the 3-space

Jörg Tellenbröker and Dietmar Kuck*

Address: Department of Chemistry, Bielefeld University, Universitätsstraße 25, D-33615 Bielefeld, Germany

Email: Dietmar Kuck - dietmar.kuck@uni-bielefeld.de

* Corresponding author

^{1}H NMR spectra including magnifications of compounds 5, 6 and 7 (600 MHz) and of compounds 8, 9, 10 and 11 (500 MHz).

^{1}H NMR spectrum (500 MHz, $CDCl_3$) of the *mixture of isomers*:

2,6,10-trinitro-4b,8b,12b,12d-tetramethyl-4b,8b,12b,12d-tetrahydrodibenzo[2,3:4,5]penta-leno[1,6-*ab*]indene (**8**)

and

2,6,11-trinitro-4b,8b,12b,12d-tetramethyl-4b,8b,12b,12d-tetrahydrodibenzo[2,3:4,5]penta-leno[1,6-*ab*]indene (**9**)

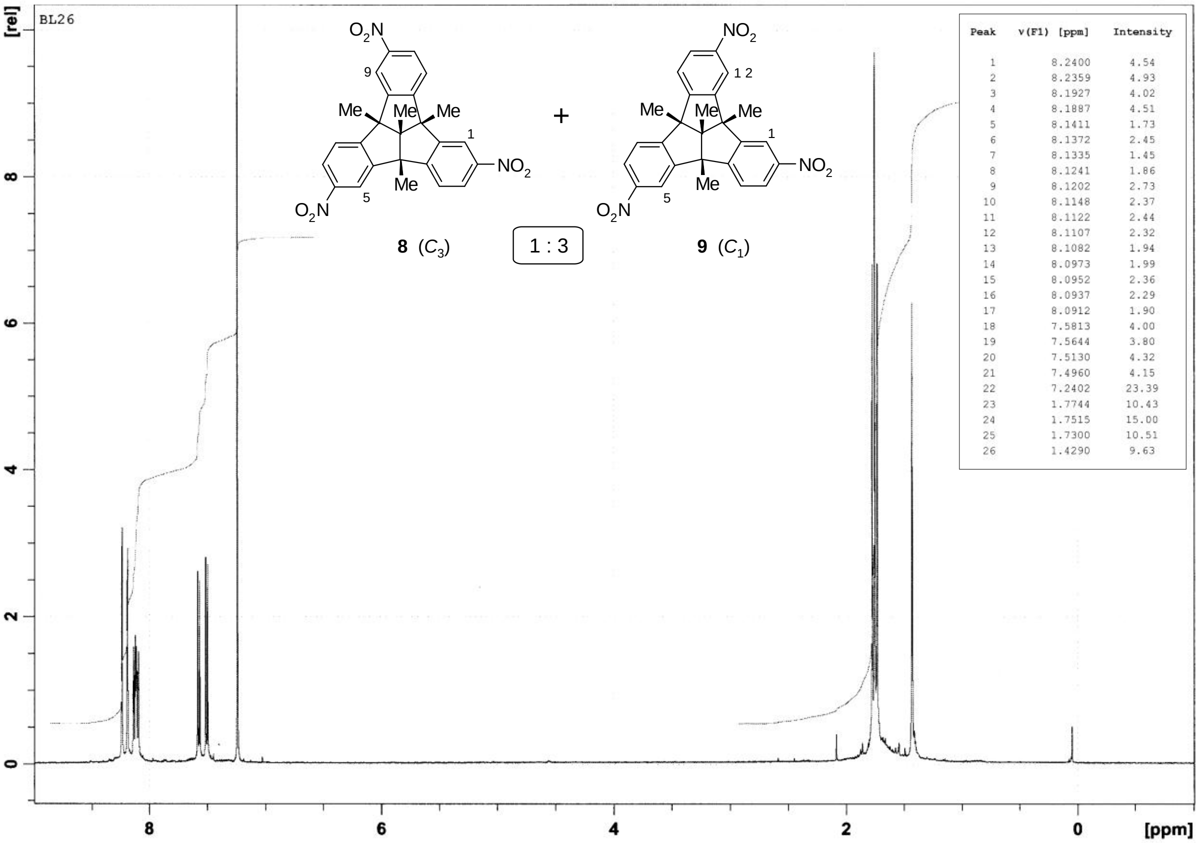

Peak	ν(F1) [ppm]	Intensity
1	8.2400	4.54
2	8.2359	4.93
3	8.1927	4.02
4	8.1887	4.51
5	8.1411	1.73
6	8.1372	2.45
7	8.1335	1.45
8	8.1241	1.86
9	8.1202	2.73
10	8.1148	2.37
11	8.1122	2.44
12	8.1107	2.32
13	8.1082	1.94
14	8.0973	1.99
15	8.0952	2.36
16	8.0937	2.29
17	8.0912	1.90
18	7.5813	4.00
19	7.5644	3.80
20	7.5130	4.32
21	7.4960	4.15
22	7.2402	23.39
23	1.7744	10.43
24	1.7515	15.00
25	1.7300	10.51
26	1.4290	9.63

Partial ^{1}H NMR spectrum (500 MHz, $CDCl_3$) of the mixture of **8** and **9**: *Aliphatic region*

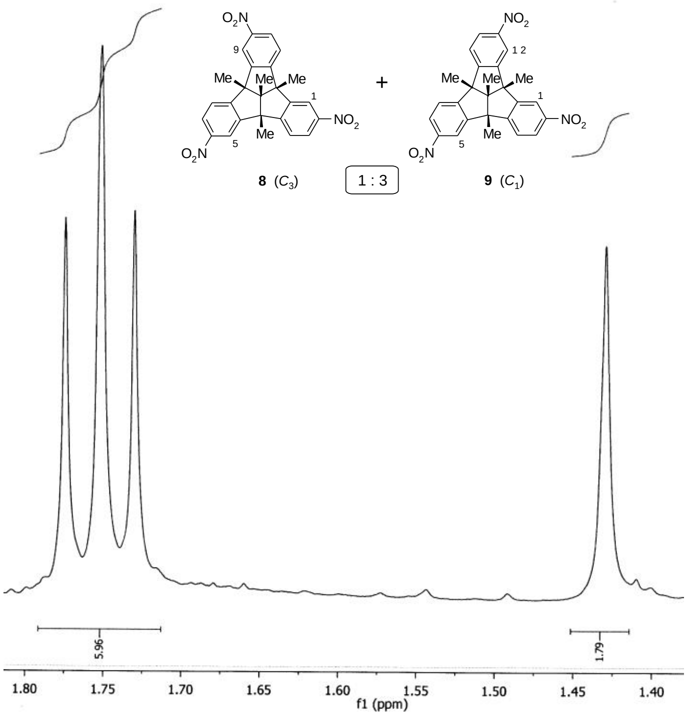

Partial ^{1}H NMR spectrum (500 MHz, $CDCl_3$) of the mixture of **8** and **9**: *Aromatic region*

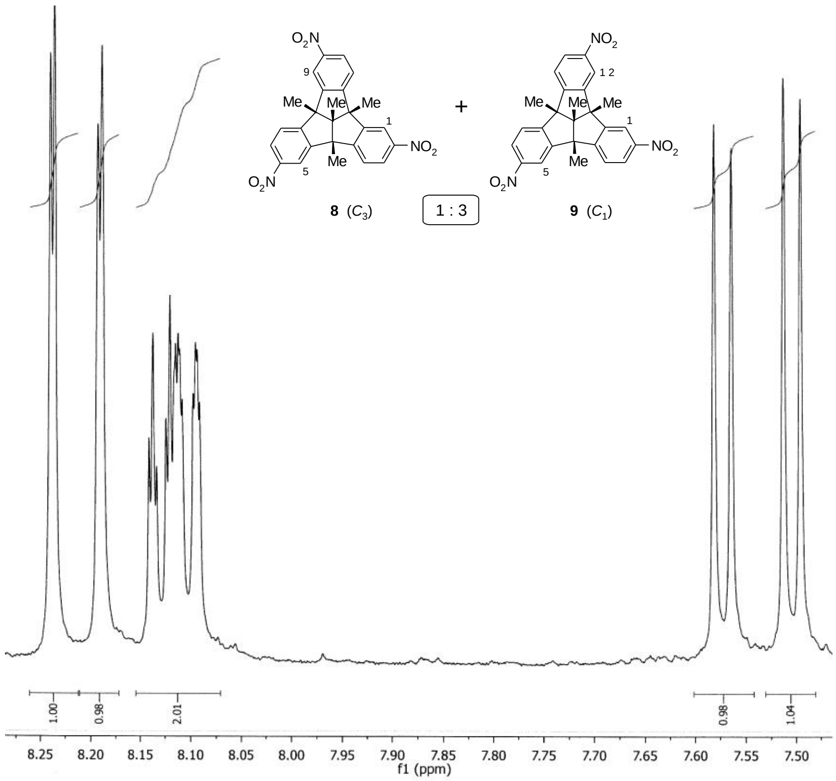

^{1}H NMR spectra (500 MHz, $CDCl_3$) of

2,6,10-triamino-4b,8b,12b,12d-tetramethyl-4b,8b,12b,12d-tetrahydrodibenzo[2,3:4,5]penta-leno[1,6-*ab*]indene (**10**)

and

2,6,11-triamino-4b,8b,12b,12d-tetramethyl-4b,8b,12b,12d-tetrahydrodibenzo[2,3:4,5]penta-leno[1,6-*ab*]indene (**11**)

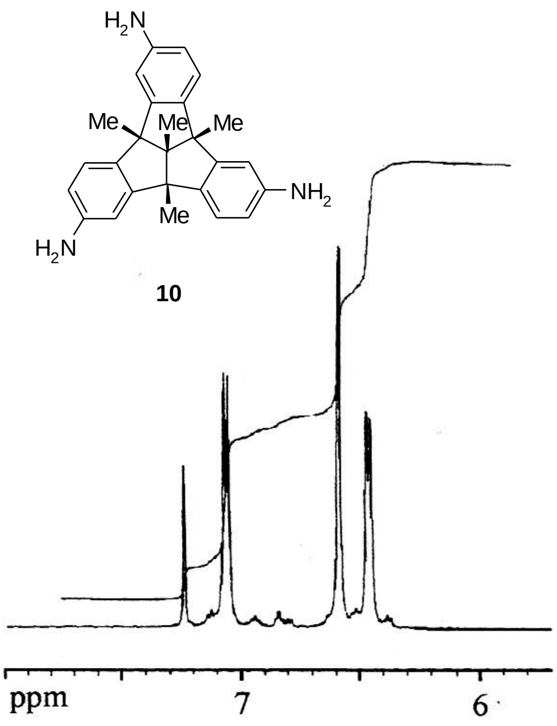

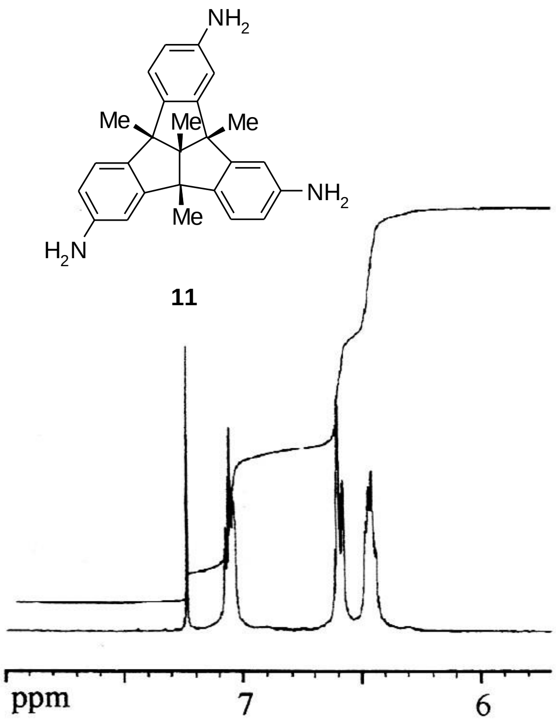

^{1}H NMR spectrum (600 MHz, DMSO-d_6) of 2,6,10-tris[(*N*-phenylcarbamoyl)amino]-4b,8b,12b,12d-tetramethyl-4b,8b,12b,12d-tetrahydrodibenzo[2,3:4,5]pentaleno[1,6-*ab*]indene (**5**)

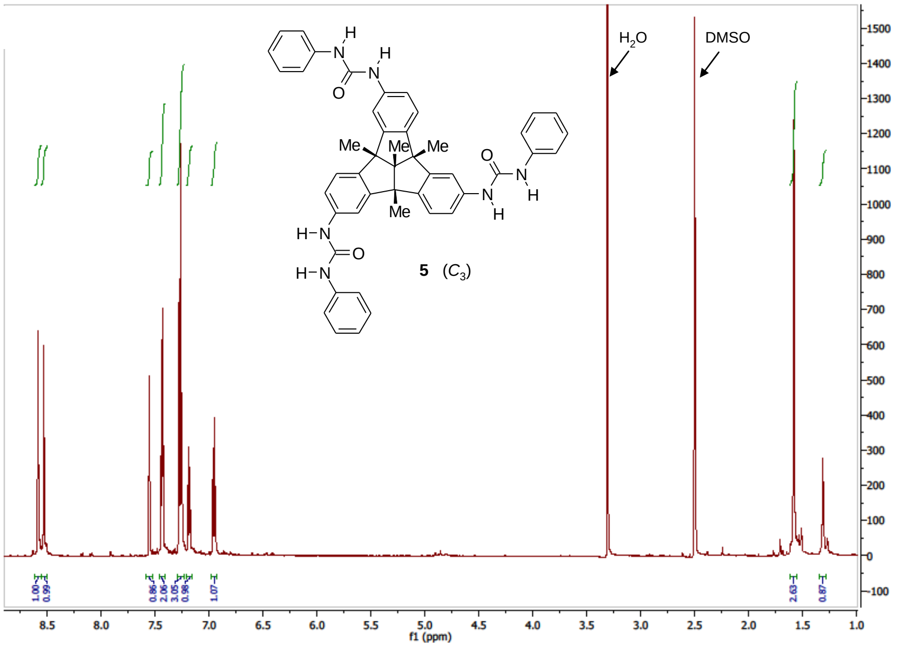

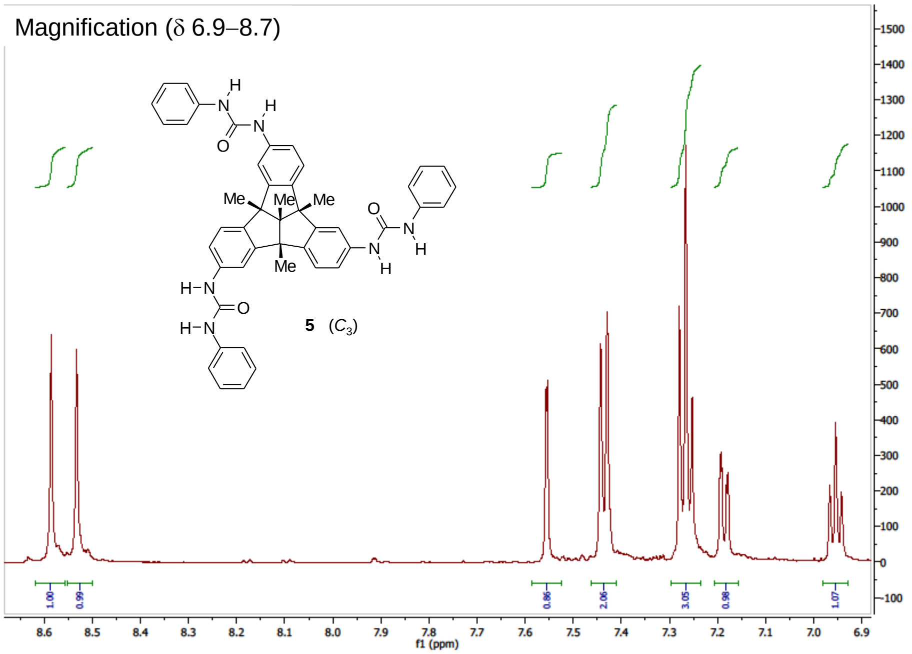

^{1}H NMR spectrum (600 MHz, DMSO-d_6) of 2,6,11-tris[(*N*-phenylcarbamoyl)amino]-4b,8b,12b,12d-tetramethyl-4b,8b,12b,12d-tetrahydrodibenzo[2,3:4,5]pentaleno[1,6-*ab*]indene (**6**)

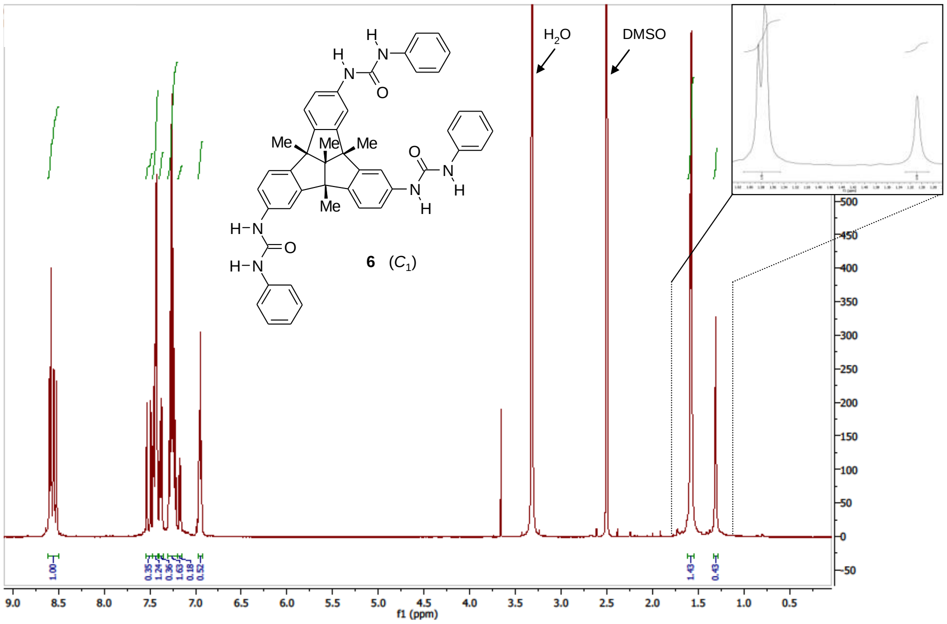

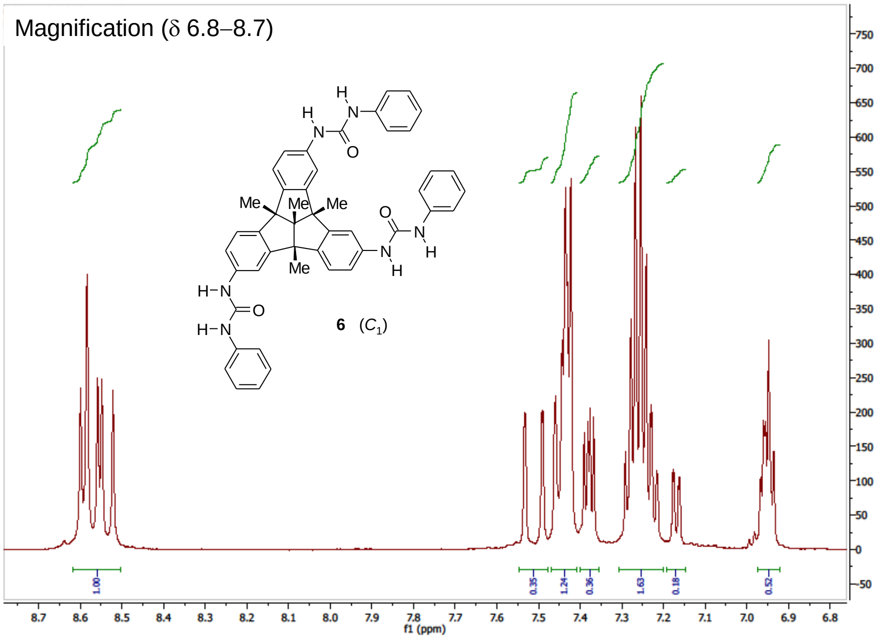

^{1}H NMR spectrum (600 MHz, DMSO-d_6) of 12d-methyl-4b,8b,12b-tris[(*N*-phenylcarbamoyl)amino]-4b,8b,12b,12d-tetra-hydrodibenzo[2,3:4,5]pentaleno[1,6-*ab*]indene (**7**)

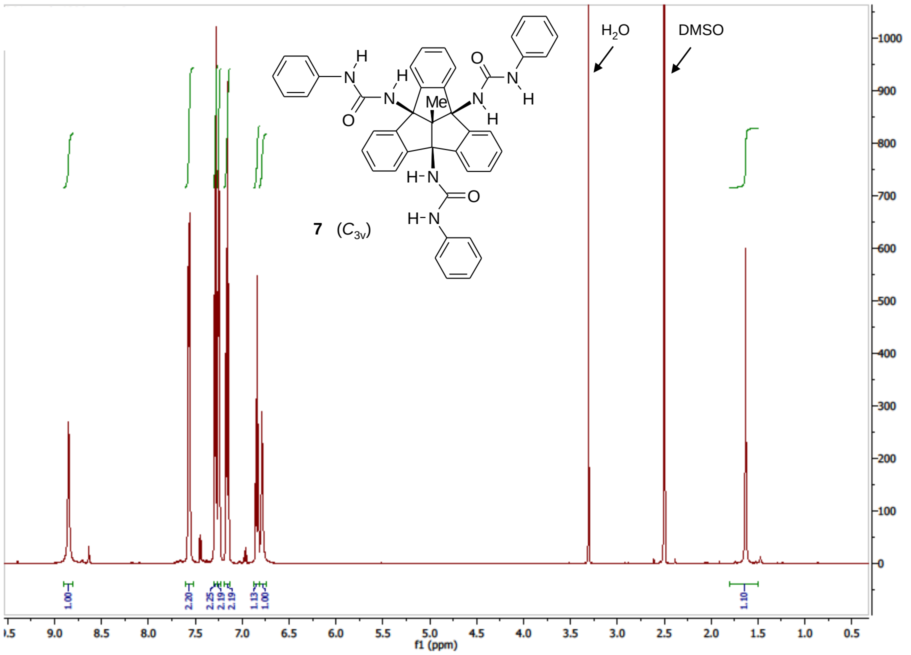

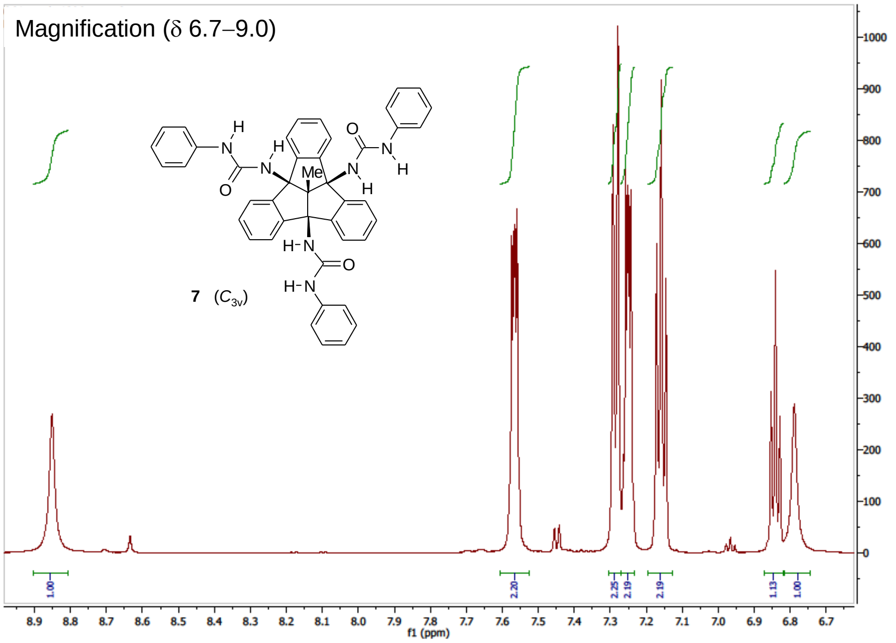

Supplement: File 1 — 1H NMR spectra including magnifications of compounds 5, 6 and 7 (600 MHz) and of compounds 8, 9, 10 and 11 (500 MHz). [file Beilstein_J_Org_Chem-07-329-s001.pdf]
